# Pramanicin Analog Induces Apoptosis in Human Colon Cancer Cells: Critical Roles for Bcl-2, Bim, and p38 MAPK Signaling

**DOI:** 10.1371/journal.pone.0056369

**Published:** 2013-02-18

**Authors:** Cagri Bodur, Ozgur Kutuk, Gizem Karsli-Uzunbas, Tayirjan T. Isimjan, Paul Harrison, Huveyda Basaga

**Affiliations:** 1 Biological Sciences and Bioengineering Program, Sabanci University, Istanbul, Turkey; 2 Department of Medical Oncology, Dana-Farber Cancer Institute, Boston, Massachusetts, United States of America; 3 Department of Chemistry and Chemical Biology, McMaster University, Hamilton, Ontario, Canada; University of Valencia, Spain

## Abstract

Pramanicin (PMC) is an antifungal agent that was previously demonstrated to exhibit antiangiogenic and anticancer properties in a few *in vitro* studies. We initially screened a number of PMC analogs for their cytotoxic effects on HCT116 human colon cancer cells. PMC-A, the analog with the most potent antiproliferative effect was chosen to further interrogate the underlying mechanism of action. PMC-A led to apoptosis through activation of caspase-9 and -3. The apoptotic nature of cell death was confirmed by abrogation of cell death with pretreatment with specific caspase inhibitors. Stress-related MAPKs JNK and p38 were both activated concomittantly with the intrinsic apoptotic pathway. Moreover, pharmacological inhibition of p38 proved to attenuate the cell death induction while pretreatment with JNK inhibitor did not exhibit a protective effect. Resistance of Bax −/− cells and the protective nature of caspase-9 inhibition indicate that mitochondria play a central role in PMC-A induced apoptosis. Early post-exposure elevation of cellular Bim and Bax was followed by a marginal Bcl-2 depletion and Bid cleavage. Further analysis revealed that Bcl-2 downregulation occurs at the mRNA level and is critical to mediate PMC-A induced apoptosis, as ectopic Bcl-2 expression substantially spared the cells from death. Conversely, forced expression of Bim proved to significantly increase cell death. In addition, analyses of p53−/− cells demonstrated that Bcl-2/Bim/Bax modulation and MAPK activations take place independently of p53 expression. Taken together, p53-independent transcriptional Bcl-2 downregulation and p38 signaling appear to be the key modulatory events in PMC-A induced apoptosis.

## Introduction

A multitude of naturally occurring or synthesized molecules has been screened for their therapeutic use in human or veterinary medicine. Having long been exploited as disinfectants and preservatives in industry, antifungal agents have not been exceptions to this. Pramanicin (PMC) is a fungal fermentation product belonging to a class of antifungal agents defined by a highly functionilized polar head and an aliphatic side chain. The previous *in vitro* analyses on cultured endothelial and leukemic T-cells confirmed its therapeutic potential both as an antiangiogenic and anticancer agent [1], [2].

Prolonged exposure to *in vitro* effective doses of PMC was previously shown to decrease cell viability and trigger caspase-dependent apoptosis [2]. PMC-induced cell death was demonstrated to be mediated by activation of both stress-related kinases p38 mitogen-activated protein kinase (MAPK) and c-Jun N-terminal kinase (JNK) while extracellular regulated kinase (ERK) activity was reported to significantly decrease upon exposure to the agent. Although a transient intracellular calcium increase was reported to follow PMC exposure in cultured pulmonary endothelial cells, this phenomenon was not synchronized with either endothelial dysfunction or cell death [1].

Physical or chemical environmental stresses including radiation, osmotic stress, and oxidative stress or cell surface receptor ligands such as growth factors, inflammatory cytokines or death receptor ligands may activate a kinase cascade which eventually stimulates stress-activated MAPKs p38 and JNK. Upstream serine/threonine kinases MAP kinase kinase kinases (MEKKs) and MAP kinase kinases (MKKs) are responsible for activation by phosphorylation and subsequent nuclear translocation of JNK and p38 [3]. Once activated, JNK and p38 are known to be capable of apoptotic modulation through activating/deactivating a series of transcription factors.

Apoptosis is a tightly regulated cell death mechanism which is activated in response to various intra-/extracellular stimuli such as oxidative stress and electromagnetic radiation that damage cellular macromolecules or signals including inflammatory cytokines and growth factors. Apoptotic execution is assumed by a family of cysteine proteases called caspases that are activated in a well-defined manner [4]. While the intrinsic pathway is triggered by apoptogenic molecule release from mitochondria, the extrinsic pathway is activated through ligand binding to death receptors on the cell surface. Whether the intrinsic or extrinsic apoptotic pathway will be in action is generally determined by the nature of the stimulus. Independent of the pathway that was activated initially, both the intrinsic and extrinsic pathways could be involved to amplify the apoptotic signal in different circumstances.

Cytochrome c release from mitochondria which marks the point of no return for intrinsic apoptotic activity is intricately regulated by interactions among Bcl-2 family of proteins. The delicate balance between the anti- and proapoptotic members of the family determines the apoptotic load within the cell. Multidomain proapoptotic Bcl-2 proteins Bax and Bak have the ability of homo-/heterooligomerization at the outer mitochondrial membrane (OMM) which causes permeabilization of the OMM and release of apoptogenic molecules including cytochrome c into the cytosol. While the antiapoptotic Bcl-2 proteins such as Bcl-2, Bcl-xL, A1 and Mcl-1 keep proapoptotic Bax/Bak sequestered preventing them from initiating OMM permeabilization, proapoptotic BH3 (Bcl-2 homology 3)-only proteins including Bim, Bid, Noxa, Bad and Puma could work to neutralize the antiapoptotic members of the family [5], [6]. Although the exact mechanisms modulating Bcl-2 proteins still remain obscure to a large extent, the balance between relative cellular amounts and activities of pro- and antiapoptotic Bcl-2 proteins is known to be strictly controlled at the gene and protein levels in healthy mammalian cells. In this regard, cellular levels of proapoptotic BH3-only Bcl-2 proteins relative to their antiapoptotic counterparts is critical to set Bax/Bak free to initiate the intrinsic apoptotic pathway.

In addition to its roles in DNA repair and cell cycle regulation, the well-known tumor suppressor p53 has been shown to be able to directly regulate apoptosis. So far, this regulation has been demonstrated to occur via modulation of Bcl-2 family protein or death receptor expressions. In addition to transcriptionally upregulating Bax, Bid, Puma, Noxa, Bak and transmembrane death receptors as a transcription factor, p53 was also reported to be capable of directly activating Bax at the protein level [7]–[13]. Recent evidence also indicates that p53 itself may also behave as a BH3-only proapoptotic protein to antagonize antiapoptotic Bcl-2 proteins as well as causing transrepression of antiapoptotic Bcl-2 gene transcription [14]–[17].

**Figure 1:**
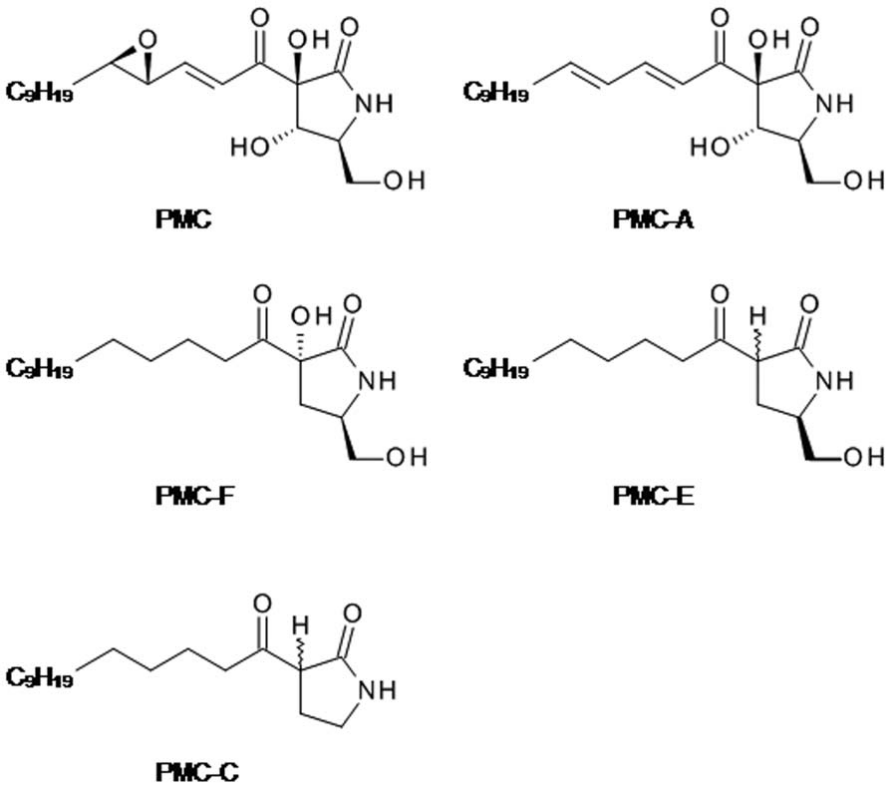
Chemical structures of PMC analogs.

In this study, we define a mechanism for intrinsic apoptotic pathway activation triggered by PMC-A, a potent PMC analog in which the epoxy group on the side chain of PMC is replaced by an alkene (Fig. 1). PMC-A mediated apoptosis through p53-independent activation of p38 and Bcl-2 downregulation in HCT-116 human colon cancer cells. Concomitantly, Bax and Bim were both accumulated in cells exposed to PMC-A with subsequent Bid truncation.

## Materials and Methods

### Cell Culture and Treatments

Wild-type (wt), p53−/−, and Bax −/− HCT116 human colon cancer cells were kindly provided by Bert Vogelstein (Howard Hughes Medical Institute, Johns Hopkins University, USA) [18], [19], cultured in McCoy’s 5A supplemented with 10% HI FBS and 100 IU/ml penicillin/streptomycin. Cultures were maintained at 37°C in a humidified 5% CO_2_ atmosphere. Ethanol (max 0.5%, v/v) was added to all control wells/plates in each experiment. Cells were collected, quantified in complete medium and seeded (100000 cells/ml) in 12-well, 6-well or 60 mm culture plates depending on the experiment. Pramanicin and analogs were added into the culture plates 36 hours later. Pre-treatments with inhibitors were done for 30 minutes prior to PMC-A treatment.

### Reagents and Analog Synthesis

#### General

Proton NMR spectra were recorded in CDCl_3_ or CD_3_OD on a Bruker AC-200 or AC-600 spectrometer and are reported as follows: chemical shift δ (ppm); number of protons, multiplicity, coupling constant J (Hz), and assignment. Residual protic solvents CHCl_3_ (δ = 7.26 ppm) and CH_3_OH (δ = 3.30) were used as the internal reference. ^13^C NMR spectra were recorded in CDCl_3_ or CD_3_OD at 50 MHz on a Bruker AC-200 and 150 MHz on a Bruker AC-600 spectrometer, using the central resonance of CDCl_3_ (δ = 77.16 ppm) as the internal reference. Infra-red spectra were recorded on a Perkin-Elmer 983G machine. Mass spectra were obtained on a Micromass Quattro Ultima (LC-ESI/APCI Triple Quadrupole Mass Spectrometer) at the chemistry department, McMaster University. The following ionization techniques were used: electron ionization (EI), and electrospray (ES). Melting points were determined on a Reichert hot stage apparatus. Optical rotations were recorded on a Perkin-Elmer (241 MC Polarimeter, λ = 589, Na lamp).

Pramanicin (PMC) and pramanicin A (PMC-A) were prepared as described previously [20]. Flash column chromatography was carried out on silica gel (SILICA GEL 60). Thin layer chromatography (TLC) was performed on pre-coated silica plates (ALUGRAM SIL G/UV_254_) and visualized by UV, vanillin or ceric ammonium nitrate solution. Aqueous solutions were saturated unless otherwise specified. The ratio of solvents in mixtures refers to the volumes used. All reactions were carried out under a nitrogen or argon atmosphere in oven-dried glassware, which was cooled under a continuous stream of nitrogen immediately prior to use unless otherwise stated. THF was distilled from sodium benzophenone ketyl, CH_2_Cl_2_ and toluene from calcium hydride, and EtOAc from potassium carbonate. Other solvents and reagents were used as supplied.

#### 3-Tetradecanoyl-1-vinylpyrrolidin-2-one (2)

Freshly distilled *N*-vinylpyrrolidin-2-one (**1**) (0.96 mL, 9 mmol) was added to NaH (1.44 g, 36 mmol) in refluxing THF (15 mL). The mixture was stirred for 30 min at 90°C, and then ethyl myristate (4.3 mL, 10.3 mmol) was added. After 3 h, the solution was warmed to room temperature, quenched with saturated aqueous NH_4_Cl, and extracted with ether. The extracts were dried (Na_2_SO_4_), filtered, and concentrated. The resulting solid was dissolved in CH_2_Cl_2_, filtered and concentrated, and purified by chromatography (CH_2_Cl_2_/EtOAc, 1∶1) to yield **2** as white crystals (3.54 g, 82%): mp 39–40°C; IR (film) 2920, 2851 (C-H, str.), 1685, 1637 (C = O, str.), 1459 (C = C, bend.), 1391, 861 cm^−1^; ^1^H NMR (CDCl_3_, 200 MHz) δ 7.01 (1H, dd, ^3^J = 9.0, ^3^J = 16.0, NCH = CH_2_), 4.46 (2H, m, NCH = CH_2_), 3.68 (1H, dd, ^3^J = 9.0, ^3^J = 6.0, COCHCO), 3.54 (1H, ddd, ^2^J = 9.0, ^3^J = 8.5, ^3^J = 5.5, CH_2_N), 3.47 (1H, ddd, ^2^J = 9.0, ^3^J = 8.5, ^3^J = 5.7, CH_2_N), 3.01 (1H, m, CH_2_CH_2_CO), 2.68 (1H, m, CH_2_CH_2_CO), 2.67 (1H, dddd, ^2^J = 13.8, ^3^J = 5.7, ^3^J = 8.5, ^3^J = 6.0, CH_2_CH_2_N), 2.12 (1H, dddd, ^2^J = 13.6, ^3^J = 8.5, ^3^J = 9.0, ^3^J = 5.5, CH_2_CH_2_N), 1.57 (2H, m, CH_2_CH_2_CO), 1.24 (20H, m, C_10_H_20_), 0.87 (3H, t, ^2^J = 6.7, CH_3_CH_2_); ^13^C NMR (CDCl_3_, 50 MHz); δ 204.2 (CH_2_ CO), 169.9 (CON), 130.0 (NCH = CH_2_), 94.5 (NCH = CH_2_), 57.3 (COCHCO), 44.0 (CH_2_N), 43.5 (CH_2_ CH_2_CO), 32.7, 30.4, 30.2, 29.8, 24.1, 23.5 (C_10_H_20_), 20.1 (CH_2_ CH_2_CH_2_), 14.9 (CH_3_); MS (EI^+^) m/z = 321 (M^⋅+^), HRMS (EI) calculated for C_20_H_35_NO_2_ (M^⋅+^) 321.2668, found 321.2679.

#### 3-Tetradecanoylpyrrolidin-2-one (3)

Compound **2** (520 mg, 1.6 mmol) was heated at 90°C in a mixture of 95% C_2_H_5_OH (35 mL) and concentrated HCl (0.5 mL). The reaction was monitored by TLC. The solution was cooled to room temperature, dried (Na_2_SO_4_), filtered, and concentrated. The resulting solid was purified by chromatography (CH_2_Cl_2_/EtOAc, 1∶1) to yield **3** as white crystals (241 mg, 50.4%): mp 73–74°C; IR (film) 3226 (N-H str.), 2920, 2851 (C-H, str.), 1716, 1671 (C = O, str.), 1468, 1375, 1278 cm^−1^; ^1^H NMR (CDCl_3_, 600 MHz) δ 5.73 (1H, brs, NH), 3.50 (1H, dd, ^3^J = 9.0, ^3^J = 6.0 COCHCO), 3.44 (1H, ddd, ^2^J = 9.0, ^3^J = 8.5, ^3^J = 5.5, CH_2_N), 3.34 (1H, ddd, ^2^J = 9.0, ^3^J = 9.0, ^3^J = 5.4, CH_2_N), 2.95 (1H, ddd, ^2^J = 17.4, ^3^J = 7.4, ^3^J = 7.5 CH_2_CH_2_CO), 2.57 (1H, ddd, ^2^J = 17.4, ^3^J = 7.2, ^3^J = 7.2 CH_2_CH_2_CO), 2.65 (1H, dddd, ^2^J = 11.0, ^3^J = 5.4, ^3^J = 9.0, ^3^J = 9.0, CH_2_CH_2_N), 2.17 (1H, dddd, ^2^J = 11.0, ^3^J = 8.5, ^3^J = 9.0, ^3^J = 5.5, CH_2_CH_2_N), 1.59 (2H, m, CH_2_CH_2_CO), 1.23 (22H, m, C_11_H_22_), 0.88 (3H, t, ^3^J = 6.8, CH_3_CH_2_); ^13^C NMR (CDCl_3_, 150 MHz) δ 205.9 (CH_2_ CO), 171.6 (CONH), 53.9 (COCHCO), 42.9 (CH_2_CO), 40.6 (CH_2_N), 32.1 (CH_3_CH_2_ CH_2_), 29.8∼29.2, 23.5 (CH_2_ CH_2_CH_2_CO), 22.9 (CH_2_ CH_2_CH_2_NH), 14.9 (CH_3_); MS(EI^+^) m/z = 295 (M^⋅+^), HRMS (EI) calculated for C_18_H_33_NO_2_ (M^⋅+^) 295.2511, found 295.2554.

#### (2R, 5S)-2-(p-Fluorophenyl)-3-oxa-1-azabicyclo[3.3.0]octan-8-one (4)

A solution of 5-hydroxymethylpyrrolidine-2-one (580 mg, 5 mmol) and *p*-fluorobenzaldehyde (810 mg, 6.5 mmol) in toluene (20 mL) containing PPTS (30 mg) was heated at reflux for 13 h using a Dean-Stark water separator. After cooling, the solution was diluted with EtOAc, washed with saturated NaHCO_3_ aqueous solution, then brine, dried (MgSO_4_), and evaporated under reduced pressure to give a brown oil. Chromatographic separation on silica gel by elution with CHCl_3_ gave **4** as a pale yellow oil (586 mg, 53%): IR (film) 2920, 2851 (C-H, str.), 1701 (C = O, str.), 1559, 1507, 1437 (aromatic C = C bend.) 1301, 1099, 821, 668 cm^−1^; ^1^H NMR (CDCl_3_, 200 MHz) δ 7.34 (2H, dd, ^3^J = 8.0, ^4^J = 5.5, H-Ph), 6.95 (2H, dd, ^3^J = 8.6, ^4^J_H-F_ = 8.6, H-Ph), 6.20 (1H, s, OCHPh), 4.15 (1H, dd, ^2^J = 7.4, ^3^J = 6.4, CH_2_O), 4.04 (1H, m, NCHCH_2_O), 3.40 (1H, t, ^2^J = 7.4, ^3^J = 7.6, CH_2_O), 2.74 (1H, ^2^J = 17.4 ^3^J = 9.6, ^3^J = 9.4, CH_2_CO), 2.47 (1H, ddd, ^2^J = 17.4, ^3^J = 3.7, ^3^J = 9.8, CH_2_CO), 2.31 (1H, dddd, ^2^J = 20.2, ^3^J = 3.7, ^3^J = 7.6, ^3^J = 7.0, CH_2_CH_2_CHN), 1.87 (1H, dddd, ^2^J = 20.2, ^3^J = 4.7, ^3^J = 9.4, ^3^J = 9.8, CH_2_CH_2_CHN); ^13^C NMR (CDCl_3_, 50 MHz) δ 178.2 (CON), 163.5 (1C, d, ^1^J_C-F_ = 244.4, CF), 129.5 (2C, d, ^3^J_C-F_ = 8.2, CHCHCF), 115.4 (2C, d, ^2^J_C-F_ = 21.5, CHCF), 86.7 (OCHPh), 71.8 (CH_2_O), 58.9 (NCH), 33.6 (COCH_2_), 23.1 (COCH_2_ CH_2_).

#### (2R, 5S)-2-(p-Fluorophenyl)-7-tetradecanoyl-3-oxa-1-azabicyclo[3.3.0]octan-8-one (5)

To a solution of protected lactam **4** (222 mg, 1 mmol) and HMPA (5 mL) in THF (15 mL), LiN(TMS)_2_ (1.0 M, 1.3 mL, 1.3 mmol) in THF was added at −78°C. The reaction mixture was stirred for 30 min, and then ethyl myristate (0.4 mL, 1.2 mmol) was added. The solution was slowly warmed to room temperature. After 3 h, the reaction mixture was quenched with saturated NH_4_Cl aqueous solution, extracted with ether, and the extract was dried (Na_2_SO_4_), and concentrated. The resulting product was purified by chromatography (hexanes/EtOAc, 2∶1) to give **5** as white crystals (233 mg, 53%) which was formed as a mixture of two diastereomers: mp 60–62°C; IR (film) 2918, 2851 (C-H, str.), 1716, 1681 (C = O, str.), 1559, 1512 (aromatic C = C bend.), 1401, 1240, 1035, 802 cm^−1^; MS (EI^+^) m/z = 431 (M^⋅+^), HRMS (EI) calculated for C_26_H_38_NFO_3_ (M^⋅+^) 43.2913, found 431.2914.

#### (5R)-5-(Hydroxymethyl)-3-tetradecanoylpyrrolidin-2-one (6)

A solution of protected lactam **5** (50 mg, 0.15 mmol) in 5 mL AcOH/THF/H_2_O (3∶7:1) was warmed at 90°C for 3 h. Benzene (30 mL) was added to the mixture and evaporated under reduced pressure. Flash chromatography was performed (EtOAc/CH_3_OH, 10∶1) to yield **6** as white crystals (22 mg, 60%) of a mixture of diastereomers: mp 73–76°C; IR(film) 3385 (NH str.), 3307 (OH, str.), 2920 (C-H, str.), 1699, 1653 (C = O, str.), 1457, 1119 cm^−1^; MS (EI^+^) m/z = 325 (M^⋅+^), HRMS (EI) calculated for C_19_H_35_NO_3_ (M^⋅+^) 325.2617, found 325.2599.

#### (2S, 5S, 7R)-7-Hydroxy-(4-fluorophenyl)-3-oxa-1-azabicyclo[3.3.0]octan-8-one (7)

Compound **6** (200 mg, 0.46 mmol) was added to a suspension of CeCl_3_.7H_2_O (5.6 mg, 0.015 mmol) in 2 mL *i*-PrOH. The suspension was mixed by bubbling air through it for 2 h at room temperature. The reaction mixture was concentrated. Flash chromatography was performed (CH_2_Cl_2_/EtOAc, 1∶1) to yield **7** as a colorless oil (120 mg, 57%): IR (film) 3384 (OH, str.), 1715, 1698 (C = O, str.), 1511, 1230, 1156 cm^−1^; [α]_D_^23^ = −0.05^0^ (c = 0.8 g/100 mL, CH_3_OH); ^1^H NMR (DMSO-d_6_, 600 MHz) δ 7.41 (2H, dd, ^3^J = 8.9, ^4^J = 5.5, H-Ph), 7.23 (2H, dd, ^3^J = 8.9, ^4^J_H-F = _8.9, H-Ph), 6.62 (1H, s, OH), 6.11 (1H, s, H-Ph), 4.28 (1H, dd, ^2^J = 8.1, ^3^J = 8.4, CH_2_O), 4.01 (1H, dddd, ^3^J = 6.8, ^3^J = 7.0,^ 3^J = 8.4, ^3^J = 6.0, NCHCH_2_), 3.51 (1H, t, ^2^J = 8.4, ^3^J = 8.1, CH_2_O), 2.75 (1H, dd, ^2^J = 13.0, ^3^J = 6.8, CH_2_CHN), 2.69 (2H, dd, ^2^J = 18.5, ^3^J = 7.2, CH_2_ CH_2_CO), 1.90 (1H, dd, ^2^J = 13.0, ^3^J = 7.0, CH_2_CHN); ^13^C NMR (DMSO-d_6_, 150 MHz) δ 209.3 (CH_2_ CO), 172.3 (CON), 162.2 (^1^J_C-F = _244.4, C-Ph), 134.7 (C-Ph), 128.3 (2C, ^3^J_C-F = _8.4, C-Ph), 115.2 (2C, ^2^J_C-F = _21.7, C-Ph), 87.7 (C-OH), 85.6 (CH-Ph), 72.2 (CH_2_O), 54.6 (CH_2_ CHN), 37.2 (CH_2_CHN), 36.2 (CH_2_ CH_2_CO), 22.6 (CH_2_CH_2_CO), 22.05∼31.25, 13.9 (CH_3_CH_2_); MS (EI^+^) m/z = 447 (M^⋅+^), HRMS (EI) calculated for C_26_H_42_FN_2_O_4_ [(M^⋅^+NH_4_)^+^] 465.3129 found 465.3126.

#### (3S, 5R)-3-Hydroxy-5-(hydroxymethyl)-3-tetradecanoyl pyrrolidin-2-one (8)

A solution of compound **7** (60 mg, 0.13 mmol) in 5 mL AcOH/THF/H_2_O (3∶7:1) was warmed at 90°C for 3 h. C_6_H_6_ (30 mL) was added to the mixture and evaporated under reduced pressure. Flash chromatography was performed (EtOAc/CH_3_OH, 10∶1) to yield **8** as white crystals (22 mg, 48%): mp 74–76°C; IR(film) 3300 (NH, str.), 3226 (OH, str.), 2995, 2849 (C-H, str.), 1688, 1650 cm^−1^ (C = O, str.); [α]_D_^23^ = −4.25° (c = 0.04 g/100 mL, CH_3_OH); 1H NMR (CD_3_OD, 600 MHz) δ 3.81 (1H, dddd, ^3^J = 4.9, ^3^J = 5.4, ^3^J = 6.3, ^3^J = 7.9, CH_2_CHN), 3.62 (1H, dd, ^2^J = 11.1, ^3^J = 4.9, CH_2_O), 3.53 (1H, dd, ^2^J = 11.1, ^3^J = 6.3, CH_2_O), 2.74 (2H, m, CH_2_CH_2_CO), 2.38 (1H, dd, ^2^J = 14.1, ^3^J = 5.4, CH_2_CHN), 2.10 (1H, dd, ^2^J = 14.1, ^3^J = 7.9, CH_2_CHN), 1.56 (2H, m, CH_2_CH_2_CO), 1.31 (3H, t, ^3^J = 7.0, CH_3_CH_2_); ^13^C NMR (CD_3_OD, 150 MHz) δ 212.2 (CO), 176.7 (CON), 84.5 (COH), 65.5 (CH_2_OH), 54.3 (CHN), 38.7 (CH_2_CO), 33.1∼30.8, 24.3 (CH_2_CH_2_CO), 14.4 (CH_3_CH_2_); MS (EI^+^) m/z = 341 (M^⋅+^), HRMS (EI) calculated for C_19_H_35_NO_4_ (M^⋅+^) 341.2617 found 341.2599.

PMC and analogs were dissolved in ethanol. McCoy’s 5A, FBS and antibiotics were from Pan Biotech GmbH (Aidenbach, Germany). Phosphatase inhibitor cocktail (PhosStop) and protease inhibitor cocktail were obtained from Roche (F. Hoffman-La Roche Ltd., Basel, Switzerland). Annexin V (FITC) was from Alexis Biochemicals (Enzo Life Sciences, Inc. Farmingdale, USA). Cleaved caspase-3, cleaved caspase-9, JNK, P-JNK, p38, P-p38, Bcl-2, Bax, Bid, Bim and β-actin antibodies were purchased from Cell Signaling Technology Inc. (Beverly, MA, USA). MAPK inhibitors SP600125 and SB203580 were from Calbiochem (La Jolla, CA, USA). Caspase inhibitors Z-VAD-FMK (general caspase inhibitor), Z-DEVD-FMK (caspase-3 inhibitor), Z-LEHD-FMK (caspase-9 inhibitor), and Z-IETD-FMK (caspase-8 inhibitor) were purchased from BD Biosciences (San Jose, CA, USA). Tris, glycine and Tween-20 were from Molekula Ltd (Shaftesbury, UK). All other chemicals were obtained from Sigma (Darmstadt, Germany).

### MTT Cell Viability Asssay

Cell viability upon exposure to PMC analogs was determined using an MTT (dimethyl thiazolyl diphenyl tetrazolium) assay kit (Roche, Mannheim, Germany) according to the manufacturer’s protocol. Briefly, HCT116 wt cells in 96-well plates were treated as indicated, and 10 µl of MTT labeling reagent was added to each well, after which the plates were incubated for 4 hours. The cells were then incubated in 100 µl of the solubilization solution for 12 hours, and the absorbance was measured with a microtiter plate reader (Bio-Rad, CA, USA) at a test wavelength of 550 nm and a reference wavelength of 650 nm. Percent viability was calculated as (OD of drug-treated sample/control OD) × 100.

### Flow Cytometry

Cell death was determined by an Annexin-V affinity assay. Wt, p53−/−, and Bax −/− HCT116 cells seeded in 12-well plates were transfected/treated as indicated, transferred to flow cytometry tubes and cells were harvested by centrifugation at 300 g for 5 minutes. Then the cells were resuspended in 1 ml of cold PBS and centrifuged again at 300 g for 5 minutes. After removal of supernatant, the cells were incubated in Annexin V buffer (140 mM HEPES, 10 mM NaCl, 2,5 mM CaCl_2,_ pH:7.4) containing 1% (v/v) Annexin V (FITC) for 15 minutes in the dark. Cells were analyzed by FACS (FACSCanto, Becton Dickinson).

### Protein Extraction and Immunoblotting

Cells were treated as indicated and harvested by centrifugation at 300 g for 30 seconds. Following resuspension in 1 ml of ice-cold PBS and transfer to 1.5-ml microfuge tubes, cells were spun at 13200 rpm for 30 seconds. The pellet was lysed by incubation for 30 minutes in 200 µl of cold cell lysis buffer containing 50 mM Tris-HCl (pH:8.0), 150 mM NaCl, 1 mM phenylmethanesulfonyl fluoride, protease and phosphatase inhibitor cocktails, and Nonidet P-40 1% (v/v). After centrifugation at 13200 rpm for 10 minutes, supernatant containing the total protein extract was removed and stored at −80°C. Protein concentrations were determined by DC protein assay (Bio-Rad, Munich, Germany). Proteins (30 µg) were mixed with loading buffer (4% SDS, 20% glycerol, 10% 2-mercaptoethanol, 0,004% bromophenol blue, 0,125 M Tris-HCl pH:6,8) and separated on 10–15% SDS-PAGE and blotted onto PVDF membranes. The membranes were blocked with 5% blocking reagent (ECL Advance, Amersham Pharmacia Biotech, Freiburg, Germany) in PBS-Tween20 and incubated with appropriate primary and HRP-conjugated secondary antibodies (Amersham Pharmacia Biotech, Freiburg, Germany) in 5% blocking reagent. After required washes with PBS-Tween20, proteins were finally analyzed using an enhanced chemiluminescence detection system (ECL Advance, Amersham Pharmacia Biotech, Freiburg, Germany) and exposed to Hyperfilm-ECL (Amersham Pharmacia Biotech, Freiburg, Germany).

### Transfections

HCT116 wt cells were transfected with Bcl-2 and Bim expression plasmids (Addgene Inc., Cambridge, MA, USA) using FuGene 6 transfection reagent (Roche, F. Hoffman-La Roche Ltd., Basel, Switzerland) according to the manufacturer’s protocol. Total protein extraction was performed after 24 hours following completion of transfection procedure for confirmation of ectopic expression. Plasmid-transfected cells were treated with PMC-A as indicated and analyzed by flow cytometry after 24 hours.

### Statistical Analysis

All the illustrated results represent one of at least three independent experiments with similar outcomes. All numerical data are presented as as means ± SD. Statistical significance of responsive differences among differentially treated or differentially transfected cell populations were assessed with unpaired or paired student’s t-test, respectively. Values of P<0.05 and P<0.01 are marked as * or **, respectively.

## Results

### PMC-A Proves More Cytotoxic Compared to Its Precursor PMC and Several Analogs against HCT116 Cells

**Figure 2:**
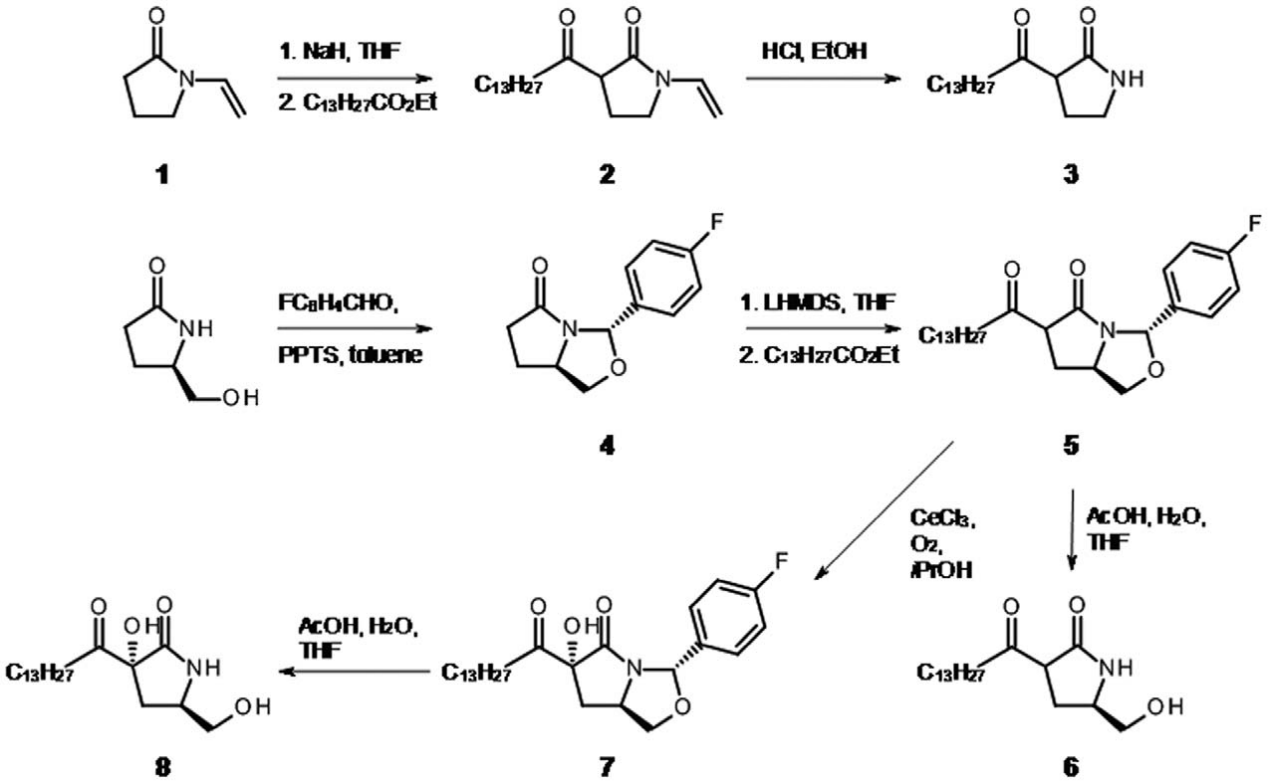
Synthesis of PMC analogs.

PMC was previously shown to selectively damage vascular endothelial cells in *in vitro* functional studies on rat aorta and dog arteries [1], [21], [22]. PMC and analogs were also demonstrated to cause cell death in cultured bovine vascular endothelial and Jurkat T leukemia cells [1], [2]. In our study, we initially investigated the previously reported natural products PMC and PMC-A [20], and then synthesized several analogs to explore structure-function relationships (Fig. 1). Thus, PMC-C was prepared from commercial *N*-vinylpyrrolidinone (**1**) by formation of the enolate, then acylation with ethyl myristate to give **2**. The vinyl protecting group was then removed with aqueous acid, giving PMC-C (**3**) as a racemic mixture at the readily epimerized stereocentre (Fig. 2).

For analogs PMC-D and –F, commercial (*R*)-5-hydroxymethylpyrrolidine-2-one was converted to the protected derivative **4**, using a literature procedure [23], then deprotonated and acylated with ethyl myristate, giving **5**. Deprotection then afforded PMC-D (**6**). Alternatively, hydroxylation of **5** with oxygen and cerium chloride as catalyst according to the method of Christoffers [24] gave **7** which was then deprotected to yield PMC-F (**8**). Use of the *p*-fluorophenyl protecting group conveniently resulted in essentially completely stereoselective hydroxylation, whereas other substituents on the benzene ring gave mixtures. The stereochemistry of **8** was established by X-ray crystallography, which revealed that the hydroxy group was *trans* to the hydroxymethyl substituent, as opposed to the *cis* relationship in pramanicin. It was therefore of interest to establish whether this hydroxy group and its stereochemistry are important for activity, and thus we included this compound in the test panel.

**Figure 3:**
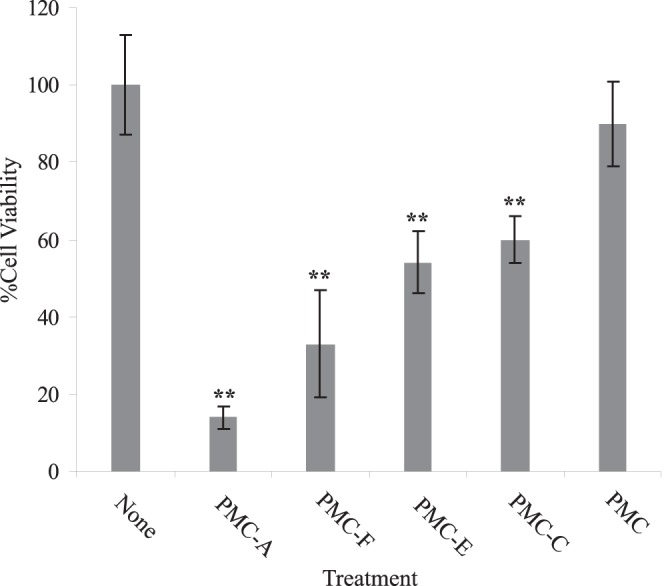
PMC-A is the most cytotoxic compound among PMC analogs. Viabilities of HCT116 cells were analyzed by MTT assay following 24 h treatment with 100 µM of each PMC analog. Wild-type HCT116 cells were assayed colorimetrically following 24 h treatment with PMC analogs. Average absorption values relative to untreated control are displayed after multiplication with 100. The results from at least 3 independent experiments were shown as means ± SD. Difference of mean values between treated and untreated samples were tested using unpaired student’s t-test; **P<0.01. Control cells were treated with solvent only. All tested analogs except the precursor PMC led to significant loss of viability/proliferation while PMC-A and -F proved most cytotoxic to cells.

We then performed a 24 hour cytotoxicity assay to search for the most potent analog against HCT116 cells. MTT reduction test which indirectly determines cell viability/proliferation by monitoring metabolic activity, revealed that 100 µM PMC caused approximately 8% decrease in cell viability (Fig. 3). However, PMC-A which possesses a C = C double bond instead of an epoxy group in its side chain, decreased the cell viability by almost 70% compared to the untreated control. Notably, analogs PMC-C, -E and –F all retained significant activity, indicating that an acylated pyrrolidinone (as in PMC-C), at most, is the key pharmacophore and that many of the substituents (epoxide, diene, C-5 hydroxymethyl group, and the C-3 and C-4 alcohol groups) are non-essential but enhance activity.

### PMC-A Induces Cell Death through Induction of Intrinsic Apoptotic Pathway

**Figure 4:**
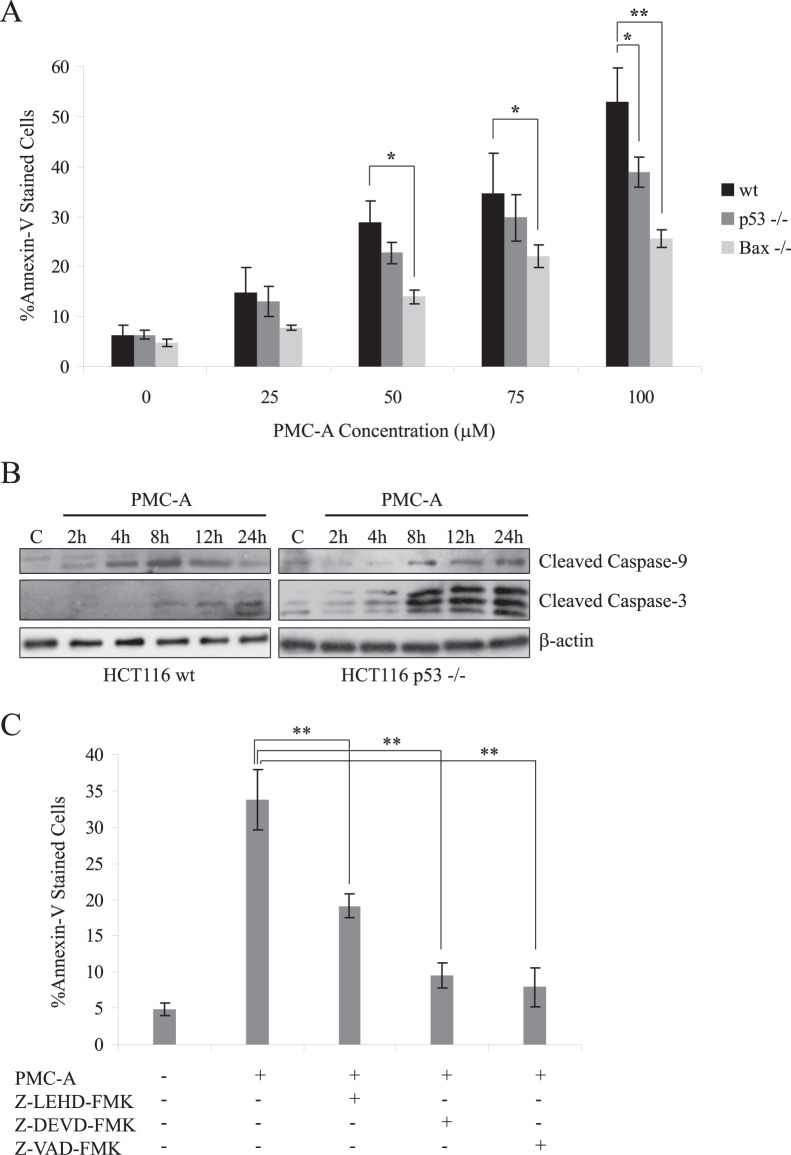
PMC-A induces dose-dependent cell death by activating intrinsic apoptotic pathway. HCT116 wt, p53−/− and Bax −/− cells were collected and analyzed by flow cytometry following 24 h treatment with indicated doses of PMC-A (A). Difference of mean values between treated and untreated cells of the same cell line were tested using unpaired student’s t-test. PMC-A caused significant dose-dependent cell death in all cell lines. Mean values of PMC-A-treated p53−/− or Bax −/− cells against those of PMC-A-treated wt cells were tested using paired student’s t-test; *P<0.05, **P<0.01. Lack of Bax expression significantly protected the cells from PMC-A induced cell death at doses of 50, 75, and 100 µM. Wild type and p53−/− HCT116 cells were treated with 50 µM PMC-A and were harvested at indicated time points, lysed and immunoblotted with anti-cleaved caspase-3, anti-caspase-9, and anti-β-actin antibodies (B). Mitochondria mediated (intrinsic) apoptotic pathway was activated as shown by cleavage of caspase-9 and caspase-3 in both cell types following the treatment. HCT116 wt cells that were pretreated with specific caspase inhibitors Z-LEHD-FMK, Z-DEVD-FMK, or Z-VAD-FMK were collected and analyzed by flow cytometry following 24 h 50 µM PMC-A treatment as well as unpretreated/untreated cells (C). Difference of mean values between pretreated and unpretreated cells were tested using unpaired student’s t-test; **P<0.01. Pharmacological inhibition of caspase-9, -3 and all caspases at once resulted in protection from PMC-A induced cell death. The results from at least 3 independent experiments were shown as means ± SD for Panel A and C. Western results from one of three independent experiments are shown. β-actin was used as loading control. Control cells were treated with solvent only.

*In vitro* effective doses of the potent PMC analog PMC-A (25–100 µM) caused cell death in a dose-dependent manner among all three HCT116 colon cancer cell lines (wt, p53−/−, and Bax −/−) as indicated by increased Annexin-V affinities in cell populations (Fig. 4A). In order to analyze cell death and determine the optimum dose to use in a 24 hour time scale, we applied flow cytometry following Annexin-V staining of cells exposed to four physiologically relevant PMC-A concentrations. Cell death induction was evident for all doses in all cell lines and increased dose-dependently. Given that, more than 50% of the wt cells were dead after 24 hour exposure to 100 µM PMC-A, we conducted the rest of the immunoblotting experiments using 50 µM dose at which approximately 70% of cells remained alive at 24 hours post-exposure.

Not surprisingly considering the wide-range modulatory activity of the tumor suppressor p53 on cell fate, lack of p53 expression proved to render HCT116 cells more resistant to PMC-A induced apoptosis at doses higher than 25 µM. Bax −/− cells on the other hand, were significantly protected from PMC-A induced cell death at all doses to a greater extent than p53−/− cells.

Critical involvement of the intrinsic apoptotic pathway that was evident by protection of Bax −/− HCT116 cells from PMC-A induced apoptosis, was further confirmed by detection of relevant caspase activities and analysis of pharmacological inhibition of these activities. We investigated the caspase activity by immunoblotting using antibodies specific to cleaved (active) forms. Caspases 9 and 3 were both activated at early hours of PMC-A treatment and these activities were sustained during a 24 hours post-exposure period in both wt and p53−/− cells (Fig. 4B). Inhibition of caspase-9, -3, or all caspases with specific chemical inhibitors significantly protected the HCT116 cells from PMC-A triggered apoptosis (Fig. 4C).

### Both Stress-related MAPKs JNK and p38 are Involved in PMC-A Induced Apoptosis: p38 MAPK Activation Appears to Be Critical

**Figure 5:**
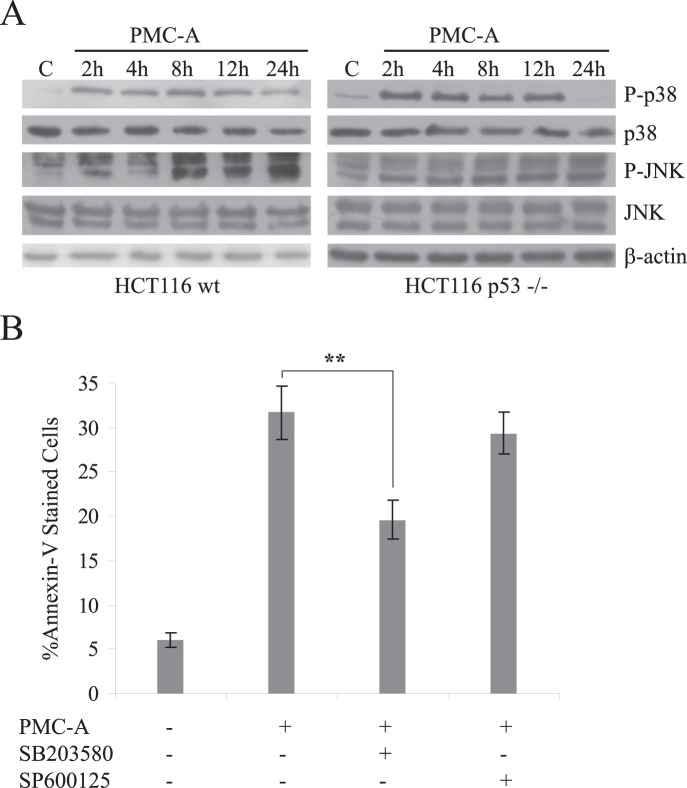
Activation of p38 is critical in PMC-A induced apoptosis. Wild type and p53−/− HCT116s were treated with 50 µM PMC-A and harvested at indicated time points, lysed and immunoblotted with anti-P-JNK, anti-JNK, anti-P-p38, anti-p38 and anti-β-actin antibodies (A). β-actin was used as loading control. Results from one of three independent experiments are shown. JNK pathway was activated at 8 h and remained active up to 24 h in both cell types while p38 was activated earlier at 2 h and maintained its activity up to 24 h in wt and up to 12 h in p53−/− cells. HCT116 wt cells that were pretreated with SB203580 or SP600125 were collected and analyzed by flow cytometry following 24 h 50 µM PMC-A treatment together with unpretreated/untreated cells (B). The results from at least 3 independent experiments were shown as means ± SD. Difference of mean values between pretreated and unpretreated cells were tested using unpaired student’s t-test; **P<0.01. Pharmacological inhibition of p38 resulted in protection from PMC-A induced cell death while JNK inhibition did not cause any significant change in cell death response. Control cells were treated with solvent only.

PMC was previously shown to induce both stress-activated MAPKs p38 and JNK, and pharmacological inhibition of these MAPKs had proved to protect Jurkat T leukemia cells from PMC induced apoptosis [2]. Similarly, we investigated the activities of JNK and p38 by immunoblotting using antibodies directed against phosphorylated active forms of these MAPKs. Our analyses in HCT116 cells revealed that PMC-A induces early activation of both JNK and p38 as indicated by increase in cellular levels of P-JNK and P-p38 (Fig. 5A). Activities of both kinases were sustained up to almost 24 hours in both wt and p53−/− cells. Moreover, pretreatment of the cells with the specific pharmacological inhibitor of p38 SB203580, but not the JNK inhibitor SP600125, prior to PMC-A exposure significantly protected the cells from apoptosis (Fig. 5B).

### Bcl-2 Dowregulation and Bim Activaton are Critical Events to Mediate PMC-A Induced Apoptosis

**Figure 6:**
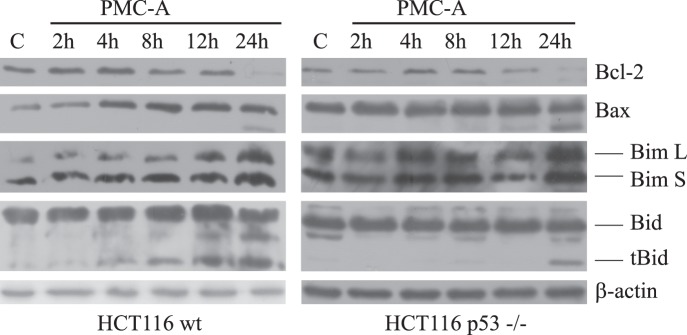
Bcl-2 downregulation and Bid processing follows Bax/Bim elevation in a p53-independent fashion in response to PMC-A. Wild-type and p53−/− HCT116 cells were treated with 50 µM PMC-A, collected at indicated time points, lysed and immunoblotted using anti-Bax, anti-Bid, anti-Bim, anti-Bcl-2 and anti-β-actin antibodies. β-actin was used as loading control. Results from one of three independent experiments are shown. Cellular proapoptotic Bax levels were upregulated as early as 2 h and sustained up to 24 h similar to the increase in L and S isoforms of another proapoptotic protein Bim in both wild type and p53−/− cells. Antiapoptotic Bcl-2 downregulation started at 8 h and culminated at 24 h post-exposure while proapoptotic Bid truncation took place in the late hours of treatment in both cell lines.

Analysis of cellular protein levels during the first 24 hours of PMC-A exposure indicated substantial changes in cellular pro- and antiapoptotic Bcl-2 proteins (Fig. 6). Early elevation of proapoptotic Bim and Bax was followed by depletion of antiapoptotic Bcl-2, and cleavage of proapoptotic Bid in response to PMC-A exposure in both wt and p53−/− HCT116 cells. Bim and Bax upregulation appeared to be initiated before 2 hours post-exposure and increased cellular Bim/Bax levels were sustained throughout the 24 hours following PMC-A treatment. Relevantly, Bax is known to be post-translationally activated by so-called activator Bcl-2 proteins Bim, Bid, and Puma to initiate OMM permeabilization as well as potentially being a target of transcription factors such as p53, SP1 (specificity protein 1), Nur77 family of nuclear receptors, and NF-κB (nuclear factor-κB) at the gene level for transactivation/-repression [5], [7], [25]–[32]. Bax/Bim upregulation was followed by Bcl-2 downregulation beginning from 8 hours post PMC-A exposure and culminating at 24 hours post-exposure. This dramatic downregulation was preceded by a transient slight increase in cellular Bcl-2 in the early hours of exposure in both wt and p53−/− cell lines. Truncation of Bid which may mark secondary involvement of the extrinsic apoptotic pathway took place as a late response to PMC-A in both cell lines. However, it should be noted that Bid cleavage was relatively delayed in p53−/− cells as cleavage product was not visible until 24 hours post-exposure.

**Figure 7:**
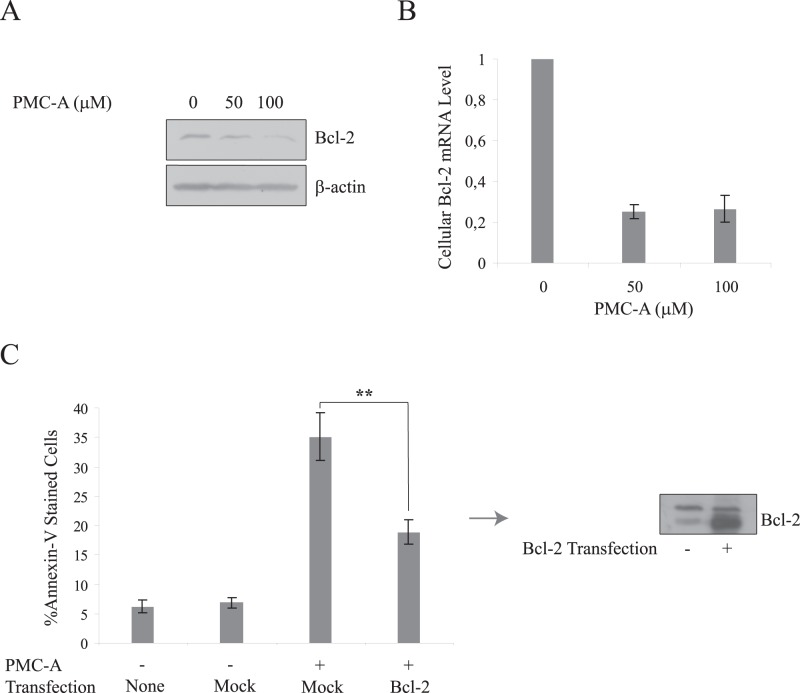
Transcriptional downregulation of Bcl-2 is critical to mediate PMC-A induced apoptosis. Wild type HCT116 cells were treated with indicated concentrations of PMC-A and harvested at 24 h post-exposure, lysed and immunoblotted with anti-Bcl-2 and anti-β-actin antibodies (A). β-actin was used as loading control. Downregulation of cellular Bcl-2 was amplified by increasing PMC-A dose. HCT116 wt cells were treated with indicated PMC-A concentrations, harvested after 24 h for total RNA extraction, cDNA synthesis and qRT-PCR analysis (B). A marginal (∼5-fold) decrease in Bcl-2 transcription was evident upon PMC-A treatment (p<0,01). HCT116 wt cells were transiently transfected with expression plasmids either carrying human Bcl-2 gene or mock vector only (C). Transfected cells were treated with 50 µM PMC-A before collection and flow cytometry analysis. The results from at least 3 independent experiments were shown as means ± SD. Difference of mean values between mock and Bcl-2-transfected cells were tested using paired student’s t-test; **P<0.01. Analysis indicates that ectopic Bcl-2 expression renders the cells more resistant against PMC-A induced apoptosis. Bcl-2 expression statuses of mock- and Bcl-2-transfected cells prior to PMC-A treatment are displayed as immunoblotting results obtained from cells that were harvested, lysed and immunoblotted with anti-Bcl-2 antibody (C). Results from one of three independent experiments are shown in immunoblotting results.

The relatively late decrease in Bcl-2 expression was dose-dependent and confirmed to take place at the transcriptional level by quantitative real-time polymerase chain reaction (qRT-PCR) analysis (Fig. 7A, B). Bcl-2 expression was decreased in PMC-A-treated cells to almost one fifth of the expression in untreated control cells. Moreover, interrogation of HCT116 cells that were transiently transfected with the human Bcl-2 gene revealed that this regulation was critical in response to PMC-A. Annexin-V binding analysis shows that cells that ectopically express Bcl-2 were significantly more resistant to PMC-A induced apoptosis (Fig. 7C).

**Figure 8:**
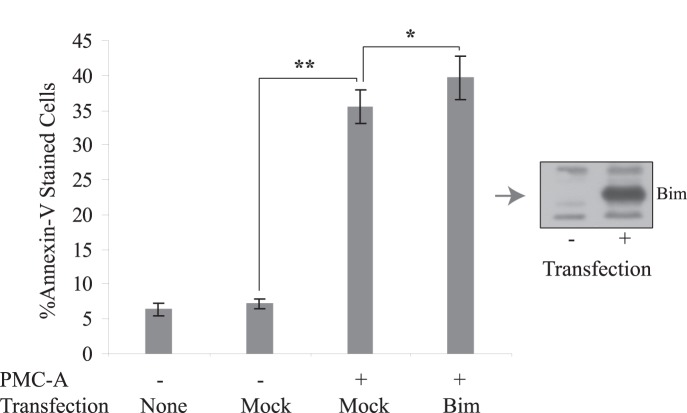
Early upregulation of Bim is important to mediate PMC-A induced apoptosis. Wild type HCT116 cells were transfected with empty, human Bim gene carrying expression plasmids. Transfected and untransfected cells were treated with or without 50 µM PMC-A and harvested 24 h following treatment and analyzed by flow cytometry. The results from at least 3 independent experiments are shown as means ± SD. **P<0.01, mean value of PMC-A-treated mock-tansfected cells against that of untreated mock-transfected was tested using unpaired student’s t-test. *P<0.05, mean values of PMC-A-treated Bim-transfected cells against that of PMC-A-treated mock-transfected were tested using paired student’s t-test. Apoptotic induction by PMC-A treatment was significantly increased in cells overexpressing Bim compared to the cells that possess endogenous Bim expression. Cells transfected with mock vector or Bim were collected, lysed and immunoblotted with anti-Bim antibody to confirm transfection.

Finally, we forced HCT116 wt cells to express BH3-only proapoptotic Bim which displayed early cellular increase in response to PMC-A (Fig. 6). Use of BimL expression vector was exploited to interrogate whether this upregulation is a contributing factor to apototic effects of PMC-A (Fig. 8). Flow cytometry analysis showed that it is possible to amplify the apoptotic response to PMC-A by ectopic Bim expression.

## Discussion

An elaborate network of pathways is required to tightly control apoptotic events in mammalian cells. Failure of surveillance/maintanence over cell fate decision could result in malignant transformation. Evasion from apoptosis as a hallmark of cancerous cells may involve dysregulation of Bcl-2 family proteins as evidence indicates that a wide variety of solid and hematopoietic tumors rely on elevated antiapoptotic Bcl-2 protein expression for survival [33]–[36]. In this view, inspection of cellular levels and activation statuses of pro- and antiapoptotic Bcl-2 proteins may provide valuable clues about anticancer drug action.As a result, interest in Bcl-2 family regulation by putative or established chemotherapetics has been increasing. The materialisation of the idea of interfering with expressions/activities of specific Bcl-2 targets in cancerous cells challenges us to develop a complete understanding of the behaviour of Bcl-2 proteins in response to different anticancer agents in various neoplasms. Although use of antisense oligonucleotides has been challenged with efficient drug delivery issues in clinical trials, significant achievements of non-peptidic small molecule inhibitors (SMI) of antiapoptotic Bcl-2 proteins are ensuring the acceleration of efforts to design Bcl-2 manipulation-based therapeutic approaches [37]–[44].

This study is the second investigation to implicate PMC analogs in anticancer activity with the aim of elucidating the basic mechanism of the induced apoptosis [2]. Although having being studied in normal vascular endothelial and leukemic T cells, pramanicin analogs was yet to be tested for anticancer action against solid cancer models. High mutation rates stemming from their deficiency in mismatch repair and the resultant aggressive behaviour associated with reported resistance against genotoxic chemotherapeutics make HCT116 epithelial colon cancer cell line an appropriate solid cancer model [45]. Cytotoxicity analysis on HCT116 cells demonstrated the potent growth inhibitory action of PMC-A as opposed to little cytotoxicity of PMC at relevant *in vitro* doses (Fig. 3). PMC-A was further confirmed to induce cell death (Fig. 4A). Early post-exposure activations of apical caspase-9 and subsequent executioner caspase-3 together with abrogation of PMC-A induced cell death upon pretreatment with pharmacological caspase-3 and general caspase inhibitors clearly indicate the apoptotic nature of the induced cell death (Fig. 4B, C).

Roughly inspecting the time course of Bcl-2-modulatory events showed early Bim and Bax upregulation followed by Bcl-2 downregulation and late truncation of Bid. Forced expression analyses of Bim and Bcl-2 ended up with identification of Bcl-2 downregulation and Bim upregulation as important mediators of PMC-A triggered apoptosis. Ectopic expression of antiapoptotic Bcl-2 prevented HCT116 cells from cell death to a large extent (Fig. 7C). On the other hand, although apoptotic promotion was significant, ectopic expression of proapoptotic Bim appeared to be less effective compared to Bcl-2 in modulating apoptosis. Given the fact that the elevation of cellular Bim and Bax precedes Bcl-2 downregulation, roles of these earlier events should not be underestimated. Bim, as an activator Bcl-2 protein, is capable of direct Bax/Bak activation upon reversible binding and known to potently antagonize all prosurvival Bcl-2 proteins which sequester Bax/Bak under normal circumstances [5], [6]. Bim may have at least 3 isoforms expressed as alternative splicing variants in mammalian cells [6], [46], [47]. Other than BimL which was ectopically expressed in HCT116 cells in this study, two other isoforms BimS and BimEL are also known to mediate apoptosis in a similar fashion to BimL [48], [49]. Immunoblotting results show that BimS is also upregulated with a similar time pattern in response to PMC-A and may also have additive roles in mediating PMC-A induced apoptosis in HCT116 cells (Fig. 6).

The Bcl-2 family of proteins were demonstrated to be regulated at the transcriptional level via transactivation or -repression through transcription factors including Mef2, Nur77, AP1, AP2, SP1, Myc/Max, Stat1, p53 and NF-κB [7], [10], [17], [26], [30], [50]–[60]. In addition, post-translational modifications such as phosphorylation, cleavage and proteasome mediated degradation were shown to take place in different cellular contexts [49], [61]–[63]. The time course of Bcl-2 downregulation roughly marks 8 hours post-exposure as the start point of this regulation (Fig. 6). Since caspase-9 activation precedes Bcl-2 downregulation, Bcl-2 depletion may not be the first event to lead OMM permeabilization, but apparently plays a substantial role in amplification of the apoptotic response. In this view, early upregulation of Bim and Bax appear to initiate caspase cascade activation through OMM permeabilization. Further investigation of PMC-A induced Bcl-2 downregulation by qRT-PCR indicated that expression of Bcl-2 was significantly repressed at the transcriptional level (Fig. 7B). Investigation of the roles of stress-activated kinases revealed that pretreatment with specific p38 pharmacological inhibitor proved to abrogate PMC-A mediated Bcl-2 downregulation while JNK inhibition did not affect the induced modulations on Bcl-2, Bim or Bax (Fig. S1). Among the transcription factors, NF-κB, SP1, p53 and AP-2 (activating protein-2) which all were previously shown to be activated by p38 kinase activity in various cellular scenarios were implicated in direct transcriptional regulation of Bcl-2 in stressed cancer cells [17], [54], [55], [60], [64]–[67]. While NF-κB and SP1 are generally implicated in transactivation of the BCL-2 gene, p53 and AP-2a were demonstrated to be responsible for transcriptional repression of Bcl-2. Relevant to our analysis, knockdown studies and further interrogation of nuclear import and activation statuses of aforementioned apoptotic modulator transcription factors, especially AP-2a, could provide further insights into the anticancer mechanism of action of PMC analogs at the nuclear level.

Bid cleavage is a well-known apoptotic event linking the extrinsic apoptotic pathway to the intrinsic apoptotic route to amplify the apoptotic signal. Procaspase-8 is activated through a sequence of events including recruitment of adaptor molecules, homooligomerization and autocatalytic cleavage upon engagement of death receptors on the cell surface [4]. Active caspase-8 which stands at the apex of the extrinsic apoptotic route contributes to procaspase-3 processing and synchronously activates BH3-only protein Bid by cleavage to initiate OMM permeabilization. Our analysis shows that Bid cleavage took place at fairly late hours following PMC-A treatment in HCT116 cells (Fig. 6). Late involvement of Bid cleavage and failure of caspase-8 inhibitor pretreatment to blunt PMC-A mediated apoptosis, makes us speculate that the extrinsic apoptotic pathway is activated secondary to the intrinsic apoptotic route (Fig. S2). Indeed, rather than being driven by a death receptor binding, such a secondary involvement of the extrinsic apoptotic route could have taken place as a result of cleavage of procaspase-8 by caspase-3 which was already activated by the intrinsic apoptotic pathway at the very beginning of PMC-A exposure [68].

Although it would be tempting to argue that PMC-A mediated Bcl-2 family regulation is related with tumor suppressor p53 signaling, all the above mentioned modulations were also apparent, at least at the protein level, in p53-null cells. However, it should be noted that Bid cleavage was visible only at 24 h post-exposure in cells lacking p53 expression while in wt cells the cleavage was evident as early as 8 hours after PMC-A treatment. This quicker response in the involvement of the extrinsic apoptotic pathway in wt cells could possibly be related with p53-dependent death receptor gene transactivation [69]. Although Bax is known to be a direct target of p53 transcription factor, the time course of cellular Bax elevation was similar in both wt and p53−/− cells upon PMC-A treatment (Fig. 6). Similarly, both Bim upregulation and Bcl-2 downregulation followed almost identical patterns independently of p53 expression in cells.

In brief, this study demostrates that PMC analogs display chemotherapeutic potential in addition to their previously shown potential against *in vitro* hematopoietic malignancy models. We define a p53-independent modulation of the Bcl-2 family of proteins as the mediator of PMC-A induced apoptosis. PMC-A rapidly induces the intrinsic apoptotic pathway through upregulation of Bax and Bim which is followed by a dramatic Bcl-2 downregulation and Bid truncation. The central role of the intrinsic apoptotic pathway was further confirmed by the protective effects of Bcl-2 overexpression and caspase-9 inhibition prior to PMC-A treatment. In addition to shedding light upon the apoptotic action mechanism of PMC-A, these results could also prove invaluable for tailoring therapeutic approaches against solid cancers. By revealing Bcl-2 downregulation as the basic mechanism of action of PMC-A to promote apoptosis, this study also provides supporting evidence for justification of clinical trials based on antisense/SMI strategies directed against Bcl-2 protein in various cancers.

## Supporting Information

Figure S1 **Pre-treatment with specific p38 inhibitor reverses PMC-A mediated Bcl-2 downregulation.** HCT116 wt cells were pretreated with indicated concentrations of JNK (SP600125), p38 MAPK (SB203580) or IKK inhibitors for 30 min, treated with 50 µM PMC-A, collected at 4 h (Bax/Bim) or 24 h (Bcl-2), lysed and immunoblotted using anti-Bax, anti-Bim, anti-Bcl-2 and anti-β-actin antibodies. β-actin was used as loading control. Results from one of three independent experiments are shown. PMC-A induced depletion of cellular Bcl-2 was restored upon pharmacological inhibition of p38MAPK in a dose-dependent manner. PMC-A induced Bim/Bax elevation was not affected with any of the three inhibitors used.
(PDF)

Figure S2 **Pre-treatment with specific caspase-8 inhibitor does not cause significant protection from PMC-A induced apoptosis.** HCT116 wt cells that were pretreated with 10 µM. Z-IETD-FMK were collected and analyzed by flow cytometry following 24 h 50 µM PMC-A treatment together with unpretreated/untreated cells. The results from at least 3 independent experiments were shown as means ± SD. Difference of mean values between pretreated and unpretreated cells were tested using unpaired student’s t-test. Z-IETD-FMK pretreatment did not cause a significant protection from PMC-A induced apoptosis at P = 0.05 level. Control cells were treated by solvent only.
(PDF)
