# Supplementary material for: Pramanicin Analog Induces Apoptosis in Human Colon Cancer Cells: Critical Roles for Bcl-2, Bim, and p38 MAPK Signaling
Source: PLoS One. 2013 Feb 18;8(2):e56369. doi: 10.1371/journal.pone.0056369 (PMC3575438; doi:10.1371/journal.pone.0056369)
Supplement: Figure S1 — Pre-treatment with specific p38 inhibitor reverses PMC-A mediated Bcl-2 downregulation. HCT116 wt cells were pretreated with indicated concentrations of JNK (SP600125), p38 MAPK (SB203580) or IKK inhibitors for 30 min, treated with 50 µM PMC-A, collected at 4 h (Bax/Bim) or 24 h (Bcl-2), lysed and immunoblotted using anti-Bax, anti-Bim, anti-Bcl-2 and anti-β-actin antibodies. β-actin was used as loading control. Results from one of three independent experiments are shown. PMC-A induced depletion of cellular Bcl-2 was restored upon pharmacological inhibition of p38MAPK in a dose-dependent manner. PMC-A induced Bim/Bax elevation was not affected with any of the three inhibitors used. (PDF) [file pone.0056369.s001.pdf]

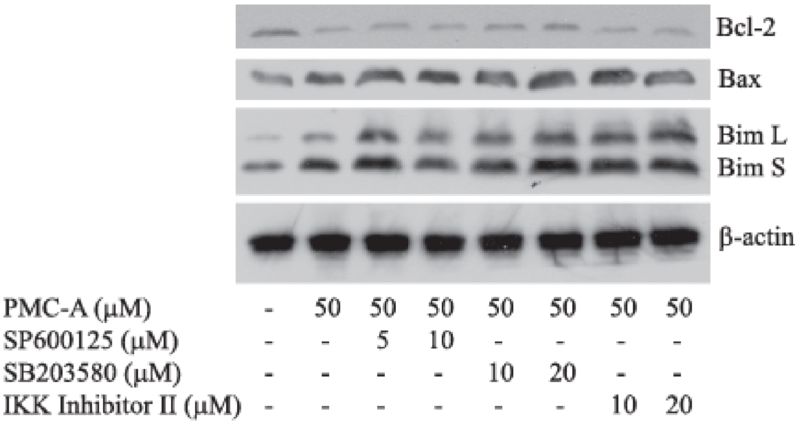

Figure S1. Pre-treatment with specific p38 inhibitor reverses PMC-A mediated Bcl-2 down-regulation. HCT116 wt cells were pretreated with indicated concentrations of JNK (SP600125), p38 MAPK (SB203580) or IKK inhibitors for 30 min, treated with 50 μM PMC-A, collected at 4h (Bax/Bim) or 24h (Bcl-2), lysed and immunoblotted using anti-Bax, anti-Bim, anti-Bcl-2 and anti-β-actin antibodies. β-actin was used as loading control. Results from one of three independent experiments are shown. PMC-A induced depletion of cellular Bcl-2 was restored upon pharmacological inhibition of p38MAPK in a dose-dependent manner. PMC-A induced Bim/Bax elevation was not affected with any of the three inhibitors used.
