# Supplementary material for: Pramanicin Analog Induces Apoptosis in Human Colon Cancer Cells: Critical Roles for Bcl-2, Bim, and p38 MAPK Signaling
Source: PLoS One. 2013 Feb 18;8(2):e56369. doi: 10.1371/journal.pone.0056369 (PMC3575438; doi:10.1371/journal.pone.0056369)
Supplement: Figure S2 — Pre-treatment with specific caspase-8 inhibitor does not cause significant protection from PMC-A induced apoptosis. HCT116 wt cells that were pretreated with 10 µM. Z-IETD-FMK were collected and analyzed by flow cytometry following 24 h 50 µM PMC-A treatment together with unpretreated/untreated cells. The results from at least 3 independent experiments were shown as means ± SD. Difference of mean values between pretreated and unpretreated cells were tested using unpaired student’s t-test. Z-IETD-FMK pretreatment did not cause a significant protection from PMC-A induced apoptosis at P = 0.05 level. Control cells were treated by solvent only. (PDF) [file pone.0056369.s002.pdf]

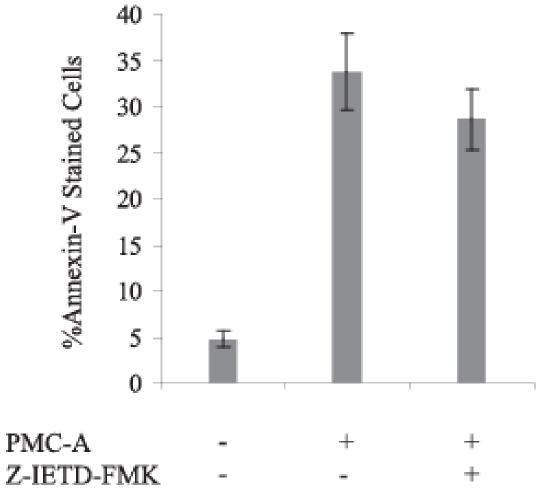

Figure S2. Pre-treatment with specific caspase-8 inhibitor does not cause significant protection from PMC-A induced apoptosis. HCT116 wt cells that were pretreated with 10 µM Z-IETD-FMK were collected and analyzed by flow cytometry following 24h 50 µM PMC-A treatment together with unpretreated/untreated cells. The results from at least 3 independent experiments were shown as means ± SD. Difference of mean values between pretreated and unpretreated cells were tested using unpaired student's t-test. Z-IETD-FMK pretreatment did not cause a significant protection from PMC-A induced apoptosis at P=0.05 level. Control cells were treated by solvent only.
